# Factors influencing the opinion of individuals in determining tumour spread after biopsy

**DOI:** 10.1186/1756-0500-4-548

**Published:** 2011-12-20

**Authors:** Tayyaba Z Ansari, Adnan A Zaidi, Asra Parekh, Owais Iqbal, Nehal Masood, Ahmed Mateen, Tehseen Fatima

**Affiliations:** 1 Aga Khan University Hospital, Stadium Road, P.O. Box 3500, Karachi 74800, Pakistan; 2 Shaukat Khanum Cancer Memorial Hospital, Main Clifton Road, Clifton, Karachi, Pakistan; 3 Karachi Institute of Radiotherapy and Nuclear Medicine (KIRAN), Gulzar-e-Hijri, K.D.A Scheme-33, Karachi, Pakistan

**Keywords:** Biopsy, Belief, Tumour spread

## Abstract

**Background:**

People often have concerns regarding tumour spread after biopsy which leads to a delay in seeking expert medical advice. The data regarding this perception is scanty. Therefore, we conducted this cross sectional study to explore the beliefs and perceptions of individuals regarding tumour spread after biopsy and the basis of those beliefs.

**Methods:**

The survey was conducted in outpatient areas of two different tertiary care hospitals of Karachi namely Aga Khan University Hospital Karachi (AKUH) and Karachi Institute of Radiotherapy and Nuclear Medicine (KIRAN). We interviewed 600 individuals and documented their responses on a questionnaire. There were 400 responders from Aga Khan's Consulting Clinic and 100 each from Aga Khan's Oncology Clinic and KIRAN.

**Results:**

Only 50% of the respondents chose biopsy as the best test for diagnosis of cancer. The level of education was statistically significant in making this choice of answer (*p *= 0.02) only in univariate analysis. Those individuals who were involved in the work up of cancer patients irrespective of their educational status gave more intelligent answers (*p *= 0.003). The tumour disturbance after biopsy was regarded as a major factor among 127 respondents (53%) who believed that biopsy could lead to spread of tumour.

**Conclusions:**

Our study revealed that awareness regarding cancer diagnosis and biopsy is lacking among general public and it does not co-relate well with the level of formal education. These misconception and taboos need to be addressed in public seminars and in the media in order to increase the awareness which could facilitate prompt diagnosis.

## Background

Cancer is one of the leading causes of deaths around the world. Tissue biopsy is essential for the diagnosis and hence the treatment of cancer [1]. Biopsy triggers the same fear among individuals as the diagnosis of cancer itself. It had been a common observation in oncology clinic that people express their fears for biopsy considering it would lead to spread of the suspected tumour [2]. The data regarding this perception is scanty but there was one study which addressed the racial differences regarding the belief that cancer spread after lung surgery [3]. This belief was prevalent in African race and the exposure to air during surgery was considered a factor leading to spread of the tumour [3]. There has been extensive evidence in the literature that biopsy does not lead to spread of tumour despite theoretical risk associated with disruption of few tumour cells during the procedure [4-7]. Nevertheless, needle track seedling can occur [8-16]. The frequency of needle tract seeding from larger series has been reported to be around 0.003 to 0.009% [5]. The current evidence negates the impact of this seeding on the overall prognosis and survival [7].

So, without evidence from literature we could only contemplate the reason of this perception in our social setting. Therefore, we decided to conduct a cross sectional study to explore the beliefs and perceptions of individuals regarding tumour spread after biopsy and the basis of those beliefs.

## Methods

The survey was conducted from January 2010 to March 2010. Initially, 400 individuals were interviewed for the survey but later 200 more individuals were included to have larger sample size [16,17]. The information regarding the type of cancer was also included later in the questionnaire to strengthen the survey.

The study was conducted in the outpatient area of two different tertiary care hospitals of Karachi namely Aga Khan University Hospital Karachi (AKUH) and Karachi Institute of Radiotherapy and Nuclear Medicine (KIRAN). At AKUH, general medicine and oncology outpatient clinics were included whereas at KIRAN no such distinction could be made as it is a specialized center for oncology cases only. So, 100 individuals were from AKU Oncology clinic and KIRAN each and 400 were from consulting clinics of AKUH. Such distribution was selected as major bulk of individuals comes to the consulting clinics for consultation. The two different departments were selected to assess the contrast in the opinion of patients at the oncology clinics and those at the general medicine clinics, assuming that people at the oncology clinic would have different a perspective on cancer and biopsies and perhaps better awareness. Two different centers were chosen to have a diverse opinion from the people as these 2 centers target different strata of population. The Aga Khan University Hospital is a private tertiary care center which receives referrals from all over the country with diverse social background. The KIRAN, on the other hand, is a public sector hospital which receives patients from different strata of the community.

An ERC (Ethics Review Committee) approval was taken from AKUH and a similar approval letter was granted from the Chair/Head of Department of KIRAN for conduction of the study. After informed consent, the responses were assessed on a printed questionnaire. The patients and the attendants at outpatient clinics of the departments of oncology and general medicine were requested to fill the questionnaire. All respondents had an understanding of English or Urdu for answering the questionnaire. The respondents belonged to different races and diverse familial backgrounds. People who refused to consent for participating in the study were excluded.

### Statistical analysis

The descriptive analysis was done for respondents' demographics. Chi-square test was used for Univariate analysis for determining the significance of individual categorical variables for the response to whether biopsy necessary for diagnosis of cancer and if it could lead to spread of tumour.

Binary logistic regression was applied for multivariate analysis for significance of different variables for the same response used in univariate analyses.

## Results

The survey included 600 individuals coming to the oncology and general medicine clinics. The distribution of the respondents was such that 100 were from oncology clinic of AKUH and KIRAN each and 400 were from AKU's consulting clinic.

The 334 of the respondents (55.7%) were male while 266 (44.3%) were females. There was not much difference in the education level between the two gender groups. Among males, 271 respondents (81%) were high school educated, graduates or professionals (post-graduates) in contrast to 220 females (82%). Nevertheless, the proportion of professionals differ between the two gender groups as 91 males (27.2%) were professionals as opposed to 40 females (15%) and this difference was statistically significant (*p *= 0.001).

Overall, only 50% of the respondents chose biopsy as the best test for diagnosis of cancer. The level of education was statistically significant in making this choice of answer (p = 0.02) in univariate analysis. The difference in the education level of respondents from different centers was statistically significant (p = 0.001) as well. There were 125 individuals (25%) who were professionals from AKUH survey in contrast to 6 (6%) from KIRAN. Interestingly, irrespective of the education status, people who were involved in the workup of cancer patients considered biopsy to be essential for diagnosis of cancer as opposed to those who had never been involved in such investigative work up (*p *= 0.001). The results of univariate analyses for categorical variables for determination of peoples' response whether biopsy is necessary for cancer diagnosis are shown in Table 1.

**Table 1:** Univariate Analysis for determining the Significance of biopsy necessary for Diagnosis of Cancer

	**Biopsy necessary**^**1**^		Significance
	
Variable Category	Yes	No	*p *value
	n (%)	n (%)	
**Gender**			
Male	253(88.2%)	34(11.8%)	0.78
Female	210(89%)	26(11%)	
			

**Education**			
None	12(75%)	4(25%)	
Primary to matric	55(91.7%)	5(8.3%)	0.02
Inter- to Graduation	274(86.2%)	44(13.8%)	
Professional	119(94.4%)	7(5.6%)	
			

**KIRAN v/s AKUH**			
KIRAN^2^	69(93.2%)	5(6.8%)	
AKUH^3^	394(87.8%)	55(12.2%)	0.24
			

**KIRAN + Oncology clinic v/s Med. clinic**			
			
KIRAN + Onc Clinic^4^	151(92.1%)	13(7.9%)	0.06
Medicine clinic	312(86.9%)	47(13.1%)	
			

**People involved in work up of cancer patients**			0.001
Yes	160(95.8%)	7(4.2%)	
No	303(85.1%)	53(14.9%)	
			

**People who believe that cancer is contagious**			
Yes	42(73.7%)	15(26.3%)	0.001
No	412(90.4%)	44(9.6%)	
			

^1 ^Only people with Yes or No response were included ^2 ^Karachi Institute of Radiotherapy and Nuclear Medicine

^3 ^Aga Khan University Hospital

^4 ^Oncology clinic

The difference of the center itself i.e. KIRAN versus AKUH did not affect the choice of the answer as *p *value was not significant (*p *= 0.24). The difference of the place became significant when distinction was made whether respondent was from the General medicine clinic, Oncology clinic or KIRAN. The proportion of people who chose 'NO' for biopsy as a necessary for diagnosis of cancer were more in General Medicine clinic of AKUH as compared to KIRAN and Oncology clinic of AKUH. This association approached near significance statistically (*p *= 0.06).

The education level, surprisingly, did not have much influence on the choice of answer whether or not biopsy leads to the spread of tumours, as 51 professionals (44%) believed that it does. This association of education level with the spread of tumour after biopsy belief did not reach statistical significance rather only approached it (*p *= 0.09). Overall, 213 responders (36%) had a firm belief that biopsy leads to the spread of the tumour. Though, 297 people (49.5%) negated this idea of tumour spread after biopsy yet remaining 88 people (14.7%) were uncertain and could not commit to either a yes or a no response. The location of the respondent was also not significant when it came to the question of tumour spread after biopsy. The detailed univariate analysis is shown in Table 2.

**Table 2:** Univariate Analysis for determining the Significance of Tumour spread after Biopsy

	Tumour spread after Biopsy^1^		Significance
	
Variable Category	Yes	No	*p *value
	n(%)	n(%)	
**Gender**			
**Male**	112(40.3%)	166(59.7%)	0.47
**Female**	101(43.5%)	131(56.5%)	
			

**Education**			
**None**	8(53.3.3%)	7(46.7%)	
**Primary to matric**	16(27.6%)	42(72.4%)	0.09
**Inter- to Graduation**	137(43.1%)	181(56.9%)	
**Professional**	51(44%)	65(56%)	
			

**KIRAN v/s AKUH**			
**KIRAN**^**2**^	35(47.9%)	38(52.1%)	0.25
**AKUH**^**3**^	178(40.7%)	259(59.3%)	
			

**KIRAN + Oncology clinic v/s Med. clinic**			
**KIRAN + Onc.**^**4**^	62(42.2%)	85(57.8%)	
**Clinic**			0.92
**Medicine clinic**	151(41.6%)	212(58.4%)	

**People involved in work up of cancer patients**			0.92
**Yes**	70(41.4%)	99(58.6%)	
**No**	143(41.9%)	198(58.1%)	

**People who believe that cancer is contagious**			0.02
**Yes**	33(56.9%)	25(43.1%)	
**No**	175(39.6%)	267(60.4%)	

^1^Only people with Yes or No response were included

^2 ^Karachi Institute of Radiotherapy and Nuclear Medicine

^3 ^Aga Khan University Hospital

^4 ^Oncology Clinic

As far as the contagious nature of the cancer is concerned, the majority of the respondents 85.5% (513) did not believe that cancer could spread from one person to another, however, 68 respondents (11.3%) still believed so. The believers of this notion were the ones who strongly perceive that biopsy leads to the spread of the tumour (*p *= 0.02) as shown in Table 2. Among those who believed in cancer being a communicable disease included 31 graduates and 11 professionals.

The two factors found statistically significant in multivariate analysis for determining whether or not biopsy necessary for diagnosis of tumour were previous involvement in the investigative work up of cancer patient (*p *= 0.003) and the belief in the contagious nature of the tumour (*p *= 0.001). However, the confidence interval was broad for the latter.

Similarly, the two factors that were found significant in multivariate analysis for whether biopsy leads to the spread of tumor were education status (*p *= 0.03) and the belief of people that cancer is contagious (*p *= 0.04). This is illustrated in Table 3.

**Table 3:** Multivariate analysis of biopsy necessary for cancer diagnosis and tumour spread after biopsy

	Biopsy necessary for Cancer diagnosis *p *value	Tumour spread after biopsy *p *value
Variables	95% Confidence Interval (CI)	95% Confidence Interval (CI)
**Gender**		
Male	0.65	0.35
Female	(0.28-1.5)	(0.58-1.21)

**Location **( different centers)		
AKUH	0.34	0.12
KIRAN	(0.15-1.9)	(0.27-1.17)

**Prior involvement in work up of cancer patients**		
Yes	0.003	0.81
No	(0.12-0.64)	(0.71-1.56)

**Education**		
None	0.32	0.03
Primary to matric	(0.28-1.51)	(0.29-0.95)
Inter- to Graduation		
Professional		

**Who believed cancer is contagious**		
Yes	0.001	0.04
No	(1.77-7.29)	(0.31-0.96)

Those who believed in tumour spread after biopsy considered tumour disturbance after biopsy as a major factor (53.4% of the responders) leading to its spread and estimated this risk to be around 50 to 90%. The basis of this belief in the majority (52%) was as 'people say it'. Many people considered the option of second opinion if faced a situation for biopsy test.

There were 200 individuals who mentioned the type of the tumour for their response. The type of the tumour for their response in decreasing order of frequency was Breast cancer (33%), Hematological cancers (16.5%), Gastro-intestinal cancers (15.5%), Bone cancers (9.5%) and Genito-urinary cancers (9%). The rest 16.5% of the individuals marked others for the primary site of the tumour for their response. The percentage response of those who believed in tumour spread after biopsy is tabulated in Table 4. The 7 responders had 100% belief that biopsy could lead to spread of tumour and they based this on their personal experience as they had witnessed themselves the increase in the size of tumour after biopsy. Overall, 35.5% of the individuals in our study believed in tumour spread after biopsy but only 1.2% experienced it.

**Table 4:** Percentage estimation of risk of tumour spread after biopsy

	Type of Cancer						
	n (%)						
	
Percentage risk of tumour spread post-biopsy	Breast	Hemato-logical	Gastro-intestinal	Bone	Genito-urinary	Others	Total
Less than or equal to	19	8	11	5	5	12	60
50 percent (± 50%)	(31.7%)	(13.3%)	(18.3%)	(8.3%)	(8.3%)	(20%)	(100%)
							

More than 50 to 90	2	3	4	1	3	4	17
percent (>50 - 90%)	(11.8%)	(17.6%)	(23.5%)	(5.9%)	(17.6%)	(23.5%)	(100%)
							

More than 90 to 100	4	1	2	0	0	0	7
percent (>90 - 100%)	(57.1%)	(14.3%)	(28.6%)	(0%)	(0%)	(0%)	(100%)

## Discussion

In our study, majority of the people had an educated background and this reflected the strata of the community which comes to AKUH for consultation. There was a marked difference in the level of education between two centers which reflected the non-uniformity of literacy level in most parts of our country [18,19].

Our study revealed that formal education affects our perception only to a limited degree regarding the understanding of cancer as almost 25% of the professionals believed that biopsy could lead to spread of the tumour. Thus, we observed that familial taboos and social circumstances affect more to a man's perception regarding this issue rather than education. This was fascinating as this belief could have been prevalent in some other communities of the world as well and has never been explored.

There were people with graduate and post-graduate qualifications who believed that cancer was communicable and one should not share utensils and clothing with the cancer patients for fear of contracting the disease. This was despite the fact that multiple posters were affixed in KIRAN stating 'cancer does not spread from one to another'. Some people even considered cancer as a calamity from God. So, a patient who suffers from the stigma of cancer also has to face the distorted perceptive of the society as well.

There were quite a few respondents (approximately 10%) in our study who had a very sound scientific knowledge of the subject despite no formal education. These were the people who had been attending on cancer patients and had gained true knowledge from the professionals directly. This fact was reflected to an extent in the analysis of the location as well. The people attending KIRAN and Oncology clinic of AKUH were more likely to know the technicalities of the issue as opposed to the Medicine clinic respondents. Consequently, more people from KIRAN and Oncology clinic of AKUH chose the medically and scientifically correct answers as compared to the Medicine clinic of AKUH irrespective of the education status.

Most of the people (50%) were very keen to know the right answer to the questions and to learn general facts regarding various aspects of cancer including diagnosis and management. This eagerness was more obvious in KIRAN than AKUH. This could be accounted for the fact that a different stratum of the society gets referred there and they lack formal knowledge to interpret the facts, hence, far more interested in learning from the professionals.

Many people (> 60%) exhibited their interest in attending public seminars on the topic and felt the need for educational programs on the TV and articles in newspaper regarding cancer education and awareness. This could make the society be more aware of the facts so that the people with cancer should not be looked down upon.

Though one third of the respondents believed in tumour spread after biopsy yet a very small number (1%) experienced it. The details of the circumstances in which biopsy had led to the tumour spread was lacking which raised the possibility of false co-relation. For example, tumour could have increased in size when there had been a delay in initiation of the treatment after biopsy or presence of occult metastatic disease at the time of diagnosis. So, it could be speculated that factors other than biopsy could have led to spread of the tumour in those circumstances.

This all suggested the need for public awareness programs as formal education tends to fail in altering the perception of individuals. The incidence of cancer is increasing all over the world, it is, therefore, imperative that common people should have some basic awareness and knowledge regarding this disease for better decision making, alleviation of the fear, and timely diagnosis.

## Conclusions

Our study revealed that misconception and taboos on the subject of cancer diagnosis, biopsy and spread are quite common in our society. Though we have targeted a selected group of people in our survey, this still has given us some insight regarding the need to address this problem among the general population. Awareness regarding the issue can be popularized through different forms of the media and using public seminars involving medical specialists. This study also opens up the prospects that these beliefs could be lying dormant in some other societies around the world which warrants further exploration.

## Competing interests

The authors declare that they have no competing interests.

## Authors' contributions

TZA conceived of the study, conducted the literature search, designed the study and formulated the questionnaire, performed the statistical analysis and drafted the main manuscript. AAZ acted as a mentor, drafted the questionnaire and liaised between the two institutions. AP and OIB collected the data by administering the questionnaire and entered the data in Epi-data. AP, in addition, converted the data into a SPSS file and helped in the statistical analysis and results tabulation. NM served as a supervisor and kept track of the timelines. AM got approval from his institution and participated in collaboration and co-ordination of the study. TF kindly rendered her services for doing the study at her institution. All authors read and approved the final manuscript.
